# Perspectives in Cell Cycle Regulation: Lessons from an Anoxic Vertebrate

**DOI:** 10.2174/138920209789503905

**Published:** 2009-12

**Authors:** Kyle K. Biggar, Kenneth B. Storey

**Affiliations:** Institute of Biochemistry and Department of Biology, Carleton University, 1125 Colonel By Drive, Ottawa, ON, K1S 5B6, Canada

**Keywords:** Cell cycle, microRNA, anoxia tolerance, retinoblastoma, ischemia, chromatin remodeling, *Trachemys scripta elegans.*

## Abstract

The ability of an animal, normally dependent on aerobic respiration, to suspend breathing and enter an anoxic state for long term survival is clearly a fascinating feat, and has been the focus of numerous biochemical studies. When anoxia tolerant turtles are faced with periods of oxygen deprivation, numerous physiological and biochemical alterations take place in order to facilitate vital reductions in ATP consumption. Such strategies include reversible post-translational modifications as well as the implementation of translation and transcription controls facilitating metabolic depression. Although it is clear that anoxic survival relies on the suppression of ATP consuming processes, the state of the cell cycle in anoxia tolerant vertebrates remain elusive. Several anoxia tolerant invertebrate and embryonic vertebrate models display cell cycle arrest when presented with anoxic stress. Despite this, the cell cycle has not yet been characterized for anoxia tolerant turtles. Understanding how vertebrates respond to anoxia can have important clinical implications. Uncontrollable cellular proliferation and hypoxic tumor progression are inescapably linked in vertebrate tissues. Consequentially, the molecular mechanisms controlling these processes have profound clinical consequences. This review article will discuss the theory of cell cycle arrest in anoxic vertebrates and more specifically, the control of the retinoblastoma pathway, the molecular markers of cell cycle arrest, the activation of checkpoint kinases, and the possibility of translational controls implemented by microRNAs.

## 1. INTRODUCTION 

Extreme hypoxia is central to a variety of diseases including cardiac and pulmonary dysfunction as well as tumor progression [1]. Recent insights into the molecular mechanisms of tumor growth have promised refined and effective cancer treatments. Although cancers are incredibly diverse, researchers have been searching for a small number of underlying controls, whose unregulated activity is required for the development of all cancers [2]. Many studies examine cell cycle components as key players in the uncontrollable proliferation and the progression of tumor growth [2, 3]. Cell cycle regulation in hypoxic environments, similar to tumor cores, has shown great potential to expose the secrets of cell cycle arrest [2, 3]. In light of this, there has been an overwhelming interest in the elucidation of the molecular mechanisms regulating survival in various levels of oxygen deprivation [3]. Many of these studies focus on anoxia tolerant invertebrate models, embryonic vertebrates and cell lines, failing to address the complexity of developmentally mature vertebrate systems. Studies examining the anoxic cell cycle characteristics of brine shrimp (*Artemia franciscana*) embryos, zebra fish (*Danio rerio*) embryos, nematodes (*Caenorhabditis elegans*) and fruit flies (*Drosophila melanogaster*) have shown that there are specific stages where the cell cycle can arrest during periods of anoxia; however, the exact mechanism of this arrest has not been proposed but may be dependant on developmental stage and/or species [3-7]. Although arrest pathways appear to be highly conserved throughout evolution, examination of a higher vertebrate animal model may prove to be useful in elucidating general mechanisms of vertebrate cell cycle arrest in response to hypoxia. Studies of this nature will prove useful for determining commonalities of cycle arrest utilizing wide comparative data between biologically diverse organisms. 

In this review, we first attempt to summarize the most relevant biochemical information for cell cycle progression with particular emphasis on the molecular pathways of cell cycle regulation. We then discuss this information in the context of recent studies of anoxia tolerant invertebrates and embryonic vertebrate model systems as well as the proposed mechanisms of hypoxia induced cell cycle arrest. Finally, we consider the implications of these findings in regards to future studies in the red-eared slider (*Trachemys scripta elegans*); a vertebrate organism known to facilitate metabolic rate depression in response to severe anoxia. 

## 2. THE CELL CYCLE

The cell cycle consists of a series of events up to and including cellular division. The typical eukaryotic cell cycle consists of four distinct phases: G_1_ phase, S phase, G_2_ phase, and M phase. Cells that have reversibly exited the cell cycle are stated to have entered a quiescence state, G_0_. The first Gap phase (G_1_) functions to ensure that mechanisms are in place to control proper DNA synthesis. In some instances, this phase can be delayed to ensure proper DNA replication *via *the G_1_ checkpoint [8]. This phase is then followed by a synthesis phase (S) facilitating the complete replication of DNA. After successful DNA replication, a subsequent gap phases (G_2_) ensures that DNA replication has properly occurred. If inappropriate DNA replication has occurred, the cell arrests *via *the G_2_ DNA-damage checkpoint before entering mitosis (M phase). After M-phase and successful completion of cell division, each daughter cell begins the G_1_ phase of a new cycle. Each interphase (G_1_, S and G_2 _phases) of the cell cycle has a distinct set of specialized biochemical processes that ultimately prepare the cell for the initiation of mitosis [8, 9].

Cell cycle control is implemented through a series of checkpoints that monitor and regulate the progress of the cycle. Proliferating cells cannot proceed through the cell cycle until individual checkpoint requirements have been met. Two main checkpoints exist capable of reversible arrest; these include the G_1_ checkpoint and the G_2_ checkpoint. The G_1_/S transition is a rate-limiting step known as the restriction point (R-point) characterized by Rb hyperphosphorylation and controlled through a delicate balance of mitotic and anti-mitotic extracellular signals [9, 10]. Inactivation of this Rb-E2F pathway characterizes the typical G_1_ checkpoint. Another important player in triggering the control mechanisms of both G_1_ and G_2_ checkpoints are the ATM/ATR checkpoint kinases [reviewed below].

### Retinoblastoma and the Cell Cycle

The retinoblastoma (Rb) gene was the first tumor suppressor to be discovered and as a result, has become a well-established member of cell cycle control [11, 12] First identified in its mutant form in the rare eye tumor, retinoblastoma, the Rb gene is known to be inactivated in nearly all human cancers, signifying its importance in the cell cycle regulation and the maintenance of proliferation [11-13]. The Rb family (pRb/p105, pRb2/p130 and p107) was initially envisioned as simple “on-off” switches regulating the progression of the cell cycle. However, recent studies have revealed a complex set of Rb:protein interactions and binding properties, adding to the importance of Rb in cell cycle regulation [11]. As a result, Rb has been shown to have a specialized role in cell cycle exit leading to senescence and quiescence as well as the ability to pause the cell cycle and block apoptosis [11-13]. These characteristics make Rb protein an intriguing target for analysis in the cell cycle systems of facultative anaerobiosis in which cells must temporarily inhibit mitosis to conserve energy stores. 

Cellular proliferation involves the inactivation of at least one member of the retinoblastoma family thereby leading to differential binding properties and phosphorylation specific Rb markers characterizing the G_1_/S transition [14]. The general mechanism by which the Rb family exerts its effects is through regulatory binding of the E2F family, inhibiting E2F-mediated transcription of cell cycle-dependent genes such as A and E type Cyclins [15, 16]. Phosphorylated pRb/p105 is present at relatively constant levels throughout the cell cycle; however, at the G_1_/S transition point it is sequentially phosphorylated by Cyclin D:Cycle dependant kinase (Cdk) 4/6 and Cyclin E:Cdk 2 complexes, respectively, leading to a release of E2F and allowing cell cycle progression ([11, 17, 18]; Fig. (**1**)). 

Unlike pRb/p105, which remains at relatively constant protein levels, p107 and pRb2/p130 are both dynamically regulated at the protein and post-translational level [19, 14]. Protein levels of p107 are maintained low during quiescence and are expressed at higher levels during G_1_ phase, parallel to that of pRb/p105 [16, 19]. In an expression pattern opposing p107, pRb2/p130 is maintained at lower levels in cycling cells and increases dramatically during quiescence [16, 19]. The rise in pRb2/p130 protein and mRNA levels at the quiescence stage is accompanied by a hypophosphorylated state, facilitating E2F-4 binding and its nuclear localization, resulting in the repression of genes required for re-entry into the early G_1_ phase. 

As previously mentioned, phosphorylation of Rb in the late G_1_ phase regulates passage through the R-point transition, committing cells to mitosis. By contrast, hypophosphorylated Rb represses E2F through two main mechanisms 1) by binding the E2F transactivation domain and 2) the recruitment of chromatin remodeling complexes [17]. The multiple protein interactions of pRb/p105, pRb2/p130 and p107 are largely controlled by the serine and threonine phosphorylation [20, 21]. Cycles of phosphorylation and dephosphorylation of pRb/p105 and p107 dominate within cycling cells and in contrast, cells undergoing quiescence harbor pRb2/p130:E2F-4 as the main pocket protein complex [13, 21]. The pRb2/p130:E2F-4 complex mediates the repression of cell cycle regulators including E2F members 1 through 3, Cdk 1 as well as Cyclins A and E [21]. When external mitogens signal cells re-enter the cell cycle, pRb2/p130 becomes hyperphosphorylated and, like pRb/p105 and p107, undergoes conformational changes, losing its repressive abilities [22]. Three amino acid residues located within pRb2/p130 (Serines 672, 952 and Tyrosine 401) are necessary for the disruption of E2F-4 repression [22]. In addition to phosphoregulation of the Rb family, pRb/p105 control through p300-dependant acetylation has been reported and is thought to regulate the G_1_/S transition by inhibiting kinase binding and Rb phosphorylation [23]. Thus, pRb/p105 acetylation (Lys 382) may act as an important regulatory control. 

Interest in the E2F family of transcription factors increased dramatically when it was discovered that E2F, members -1 through -5, complexed with Rb [24]. Knowledge that E2F regulated the cell cycle provided a mechanism by which Rb could repress cellular proliferation [25]. The E2F family is comprised primarily of five members, E2F-1, -2 and -3 that interact exclusively with pRb/p105 and p107, as well as E2F-4 and -5 which interact with pRb2/p130 and to a lesser degree p107 [25]. On the basis of sequence homology and functional properties, E2F-1 through -3 are potent transcriptional activators and will be referred to as ‘activating’ E2Fs. In contrast, E2F-4 and -5 are primarily involved in active repression of E2F targeted genes by recruiting pRb2/p130 and associated chromatin remodeling complexes; these E2Fs will appropriately be referred to as ‘repressive’ E2Fs throughout the remainder of this review [24]. 

### Cyclins, Cdks and Cdk Inhibitors

Cellular proliferation is initially driven from the presence of extrinsic mitogens [26]. The presence of growth factors triggers signal cascades which, exert regulatory effects within the cytoplasm. These growth factors lead to sequential activation of Cdks, the central driving force of the cell cycle [19, 26, 27]. Initial activation of Cdk 4 and 6 leads to sequential phase-specific Cdk activation, creating a hierarchal system ensuring that one phase of the cell cycle is completed before the initiation of the next ([26]; Fig. (**2**)). The ordered progression through cell-cycle phases is controlled by the sequential phosphorylation and activation of Cdks 4/6, Cdk 2, and Cdk 1 [19]. Cdk activity is regulated by several mechanisms including binding of a Cyclin regulatory subunit, post-translational modifications and the association/disassociation with Cdk inhibitors (CKIs). 

Mitogenic growth factors begin the cycle through the Ras/Raf/ERK pathway, activating transcription factors responsible for Cyclin D expression [27]. Cyclin D then assembles with Cdk 4/6 to form Cyclin D:Cdk 4/6 kinase complexes, carrying out the initial phosphorylation of pRb/p105 (residues Ser 780, Ser 795, Thr 807/11 and Thr 821) and marking the mid G_1_ phase [27, 28]. Following Cyclin D:Cdk 4/6 activation and the initial phosphorylation of Rb, Cyclin E (E1 and E2) is expressed by internal E2F signaling pathways, marks the entry into late G_1_ phase and the R-point transition [12]. Cyclin E forms an active kinase complex with Cdk 2, leading to final phosphorylation and complete inactivation of pRb/p105 (residues Ser 608, Ser 612 and Ser 567), dissociating the Rb-E2F complex and allowing the expression of genes required for S-phase entry and DNA replication [22, 29]. Soon after Cyclin E is expressed at the G_1_/S boundary, cells commit to mitosis and begin to express Cyclin A (A1 and A2). The increase of Cyclin A/Cdk 2 complex facilitates the activation of DNA replication machinery and is characteristic of the S-phase [30]. After completion of the S-phase and DNA replication, entry into G_2_ is marked by the expression of Cyclin B (B1, B2 and B3) and inactive Cyclin B:Cdk 1 complexes, in addition to the switch from Cyclin A:Cdk 2 complexes to the expression of active Cyclin A:Cdk 1. Cyclin B:Cdk 1 remains inactivate late into the G_2_ phase pending Cyclin B localization to the nucleus and Cdk 1 activation [30-32]. Targets of the Cyclin B:Cdk 1 complex include structural proteins involved in the execution and timing of mitotic events [reviewed in 26,^ 33^,^ 34^]. Similar to Cdk activation, Cyclin:Cdk complexes trigger the activation of the subsequent and as a result appears to be self-regulating, driving the cell cycle further through progression. During periods of cellular stress, the inhibition of Cyclin:Cdk complexes at the G_1_ checkpoint ultimately lead to the disruption of the cycle and inhibits the downstream activation of Cyclin:Cdk complexes. The cyclic expression of Cdks and Cyclins driving specific phases of the cell cycle allows for the use of cell cycle markers that when compared, can yield the cell cycle phase of synchronized cells ([19]; Fig. (**2**)). 

The basic framework of the cell cycle consists of Cyclin:Cdk complex formation, initiating the pre-activation of Cdks. The dependence of Cdk activity on Cyclin binding represents the primary mechanism whereby Cyclins mediate the reconfiguration of residues involved in ATP binding and the repositioning of the T-loop [10]. Beginning from quiescence, the introduction of growth factors allow for a depression of Cdk inhibitors and an increased expression of the first Cyclin present in the cell cycle, Cyclin D (D1, D2, and D3) [26]. Since Cdk 4/6 proteins are expressed at relatively constant levels throughout the cell cycle, the expression of Cyclin D is the rate-limiting step in Rb phosphorylation, even during early G_1_ when Cdk 4/6 expression is highest [35]. In addition, Cyclins play an additional regulatory role through nuclear and cytoplasmic shuttling of Cdks thereby suggesting a role where Cyclins act to regulate the cell cycle in a manner discrete from just-in-time expression ([10]; Fig. (**2**)). 

A recent study has shown that Cyclin D is upregulated in response to growth factors acting through the PI3K/AKT/mTOR pathway. As a response to the effect of mTOR on translational machinery, this study concluded that PI3K was absolutely necessary for induction of Cyclin D translation [36]. It has also been established in the literature that in response to environmental stresses, such as hypoxia, *C.elegans* enters a quiescent dauer stage effectively shutting down the PI3K/AKT/mTOR pathway and reducing translational activity [37]. Hence, the severe environmental stress response pathway seen in *C.elegans* may be mirrored in other stress tolerant organisms leading to reductions in Cyclin D expression and most likely playing a role in cell cycle arrest. 

## 3. CELL CYCLE REGULATION

### Stress Activated Checkpoint Pathways

Checkpoint pathways ensure error-free DNA replication and chromosome segregation, thereby tightly regulating cell cycle transitions and ensuring the maintenance of genomic integrity. Such checkpoints are comprised of apical signal transducing kinases such as phosphatidylinositol 3-kinase (PI3K)-like family members ATR and ATM kinases [38, 39]. These kinases regulate the distal serine/threonine signal transducing kinases, Checkpoints 1 and 2 (Chk1 and Chk2). These distal kinases regulate a diverse group of effector proteins encompassing cell cycle regulators such as cdc25 phosphatase, p53, E2F-1, Cyclin:Cdk complexes, and chromatin remodeling components controlling G_1 _and G_2 _arrest (Fig. (**3**)). 

To prevent inappropriate entry into both S and M phases, cells progressing though the G_1 _and G_2_ phases, respectively, activate the checkpoint transducing kinases ATR/ATM and Chk1/2. ATM-activated Chk2 primarily targets two critical effectors mediating the G_1 _checkpoint, cdc25a and p53 [38]. Phosphorylation of cdc25a, the primary phosphatase responsible for Cdk 2 activation, by Chk2 at residue Ser 123 leads to enhanced ubiquitination and proteasome-mediated degradation [39]. In contrast to cdc25a, p53 is phosphorylated by Chk2 at sites Ser 15 and 20, stabilizing p53 expression and leading to enhanced transcriptional activity [38]. One key gene upregulated by p53-mediated transcription is the Cdk 2 inhibitor, p21 [reviewed below]. Accumulation of p21 is capable of inducing G_1_ arrest by blocking Cyclin E:Cdk 2 activity, thereby maintaining pRb/p105 in a hypophosphorylated state and E2F repression. ATR/Chk1 activation of the G_2_ checkpoint prevents cells from entering mitosis when subject to DNA damage [40]. The key downstream target of the G_2_ checkpoint is the Cyclin B:Cdk 1 kinase complex. Activation of Cyclin B:Cdk 1 is prevented primarily through Chk1-mediated phosphorylation (Ser 216) and inhibition of cdc25c phosphatase, the activating phosphatase responsible for Cdk1 activation at the G_2_/M boundary [40]. Ultimately, the ATM/ATR checkpoint pathway mediates its effects by inhibiting Cdks, the primary motors of the cell cycle. 

Although hypoxia does not itself induce DNA damage or a typical DNA damage response, several studies have indicated the hypoxia-induced phosphorylation and activation of Chk2 in an ATM-dependant manner [41, 42]. Although the mechanisms for this arrest have not been clearly defined, hypoxia induced a rapid G_1_ arrest similar to that induced through DNA damage [41]. In addition, hypoxia activated Chk2 induces cell cycle arrest through similar mechanisms as DNA damage; this includes the activation and stabilization of p53 and targeting cdc25a for degradation. Activation of Chk2 may prove to be a crucial mechanism initiating hypoxic cell cycle arrest. 

### Regulation of cdk Complexes

Similar to the Rb family of pocket proteins, Cdks are subject to cycles of regulation *via *reversible phosphorylation. As previously mentioned, the binding of Cyclins to Cdks yield partial activation, however, complete activation requires neighboring phosphorylation of the Cdk ATP binding cleft. Activating phosphorylation of Cdks occurs on a conserved T-loop threonine residue (Thr 160 in Cdk 2, Thr 161 in Cdk 1 and Thr 172 in Cdk 4/6) mediated by the threonine kinase, Cdk-activating kinase (CAK). The phosphorylation of Cdks by CAK is antagonized by the specific phosphatase activities of KAP, which acts on monomeric Cdks after Cyclin degradation ([43]; Fig. (**4**)).

In addition to cyclin-dependent regulation of Cdk activity, other important mechanisms of regulation of Cyclin:Cdk complexes are mediated through inhibitory proteins, CKIs. These inhibitors have been classified into two families, INK4 and Cip/Kip, based on their mechanisms of inhibition [44, 45]. The INK4 family of Cdk inhibitors (p16INK4a, p15INK4b, p18INK4c and p19INK4d) has been found to regulate monomeric Cdks 4 and 6. INK4:Cdk binding overlaps the Cdk region responsible for Cyclin binding, thus blocking the formation of the Cyclin:Cdk complex and inhibiting Cdk function [26, 46]. The Cip/Kip family of inhibitors (p21, p27 and p57) inhibits both monomeric Cdks and Cyclin:Cdk complexes [47, 48]. Binding of the Cip/Kip family to Cdks can completely shut down the active Cyclin:Cdk complex through the insertion of a small 3^10^-helix into the Cdk catalytic cleft, antagonizing the interactions of ATP substrate [49]. 

The Cip/Kip inhibitors, p21 and p27, are both expressed during quiescence and are thought to be responsible for the characteristically low levels of Cyclin:Cdk complexes [19]. A decrease in p27 and p21 allows for increased formation of active Cyclin:Cdk complexes leading to phosphorylation of Rb and re-entry into the cell cycle. The maintenance of quiescence is self-sustaining, through the combinatory effects of CKIs, in the absence of mitogenic stimuli. These stimuli eventually lead to the active phosphorylation of Cdks and the decrease of CKIs [19, 50]. 

Several studies have indicated that hypoxia-inducible factor 1α (HIF-1α) plays an essential role in the adaptive response of cells to hypoxia [51]. In hypoxic environments, HIF-1α has a recognized role in the reorganization of glycolysis and has recently been indicated in the cessation of cellular proliferation through the induction of CKIs. A recent study by Horree has suggested that an increase in HIF-1α transactivation activity increases the expression of p27 [52]. Complimentary studies using HIF-1α null murine embryonic fibroblasts identified p27 as having a role hypoxia-induced cell-cycle arrest and that this expression is indeed HIF-1α dependant [53]. The HIF pathway may play an important role in connecting typical hypoxia response to the induction of cell cycle arrest.

### Retinoblastoma and Cell Cycle Arrest

Cells rely on several main pathways to regulate and maintain a quiescent state. These pathways converge on the necessity of pRb2/p130 and CKIs [47, 48, 53, 54, 55]. The Rb family member pRb2/p130, recruits the repressive E2F-4 to E2F target promoters and facilitates binding to chromatin-remodeling complexes [54]. This chromatin remodeling blocks the transcription of many positive regulators of the cell cycle. In addition, quiescence may be marked through site specific phosphorylation of pRb2/p130. Several studies have demonstration that the Loop region in the B pocket of pRb2/p130 harbors residues that are responsible for G_0_/G_1_ phosphorylation of pRb2/p130 at sites Ser 948, Ser 966, Ser 962, and Ser 982 in a GSK3β dependant manner [55]. This region, however, is not essential for those functions of pRb2/p130 associated with the ability to block the cell cycle progression and include the interactions with E2F-4, Cyclins A and E, and the LXCXE-containing proteins; nevertheless this phosphorylation pattern does provide a novel indicator of cellular quiescence [55]. 

Heterochromatin is composed of genomic DNA tightly packed by histones and non-histone proteins [56, 57]. Dynamic changes to chromatin structure prevent the access of transcription factors, such as E2F, to nucleosomal DNA. At least two primary mechanisms can be used to remodel chromatin structure. One mechanism involves changing the location and conformation of the nucleosomes through the use of ATP-dependent protein complexes such as SWI/SNF [58-62]. The second mechanism involves covalent modifications of histone N-terminal histone tails that protrude from the chromatin structure [57, 63]. Studies examining the role of Rb:E2F mediated cell cycle arrest have identified key Rb interactions with chromatin remodeling factors [64]. Rb-mediated chromatin remodeling effectively represses E2F transcriptional activity [64]. The particular associations between Rb and chromatin remodeling factors have been found to be dependent on the type of cell cycle exit [56]. 

General mechanisms of Rb-mediated chromatin remodeling include the recruitment of ATP-dependent chromatin remodeling complexes. One particular complex member associated with Rb during cell cycle arrest is the SWI/SNF complex and its central subunit, Brahma (BRM) [65, 66]. These chromatin remodeling complexes use the energy derived from ATP hydrolysis to alter the chromatin structure. The presence of the conserved Rb-binding motif, LXCXE, in BRM suggests that this may be the initial chromatin remodeling factor which binds to the Rb pocket domain, and prepares the nucleosomes for heterochromatin formation [65]. 

Following BRG binding and nucleosome sliding, stable repression of Rb:E2F is achieved through covalent modifications of protruding histone N-terminal tails [66]. Deacetylation of histones is mediated through the recruitment of histone deacetylases (HDACs) *via *the Rb associated protein RbAp48. The primary site of deacetylation during cell cycle exit is K9AcH3, which later becomes methylated and a site of HP1 binding [67]. Following K9AcH3 deacetylation, the next set of covalent histone modifications includes the methylation of lysine residues by histone methyltransferase (HMTase). The HMTase, Suv39H1, binds to Rb through the LXCXE binding motif and methylates lysine 9 of histone H3 [62, 67, 66]. Transcriptional repression of E2F through heterochromatin formation is also promoted through the actions of HP1 binding methylated K9H3 histones [66]. Binding of HP1 mediates higher levels nucleosome structure facilitated by HP1 dimerization, leading to multiple histone recruitment and tight chromatin packing [68]. Although these chromatin modifications are well cited in the literature, there are slight modifications to these complexes in cells undergoing a short-term pause in the cell cycle (G_1_ arrest) and those entering a prolonged reversible exit (quiescent) ([63]; Fig. (**5**)). 

Quiescent transitions are controlled in a reversible manner, mediated in part by the competing actions of histone acetyltransferases (HATs) and HDACs on the histones associated with the Rb:E2F complex. Although the general model of chromatin remodeling complexes have been characterized for cell cycle arrest, specific complexes characterizing prolonged and reversible cell cycle exit are only beginning to be explored. Chromatin modifying factors that have been found to interact with pRb2/p130 during quiescence include components of the drosophila Rb-E2F and Myb (dREAM) complex initially discovered in arrested drosophila embryos, and the methyl-histone lock, L3MBTL1 [63, 69]. These complexes contain additional quiescent specific factors which are thought to regulate quiescence by compacting nucleosome structures in a manner that is dependent on mono- and di-methylation of histone H3K9 [62]. Recruitment of L3MBTL1 allows the binding of at least two nucleosomes simultaneously through the recognition of N-terminal histone modifications [62]. This raises the possibility that dREAM may promote specific and reversible heterochromatin structure through the recruitment of L3MBTL1, locking methylated histones ([63]; Fig. (**5**)). 

In accordance with quiescence-dependent Rb expression, pRb2/p130:E2F-4 but not pRb/p105:E2F-1 interacts with members of the dREAM complex [70]. During quiescence, the dREAM complex binds to more than 800 promoters and is found in association with E2F target gene promoters [70]. Components of the dREAM complex include LIN9, F25965 (LIN37), LOC91750 (LIN52) and Tesmin [62]. Although the functions of F25965 and LOC91750 have not yet been characterized, knock-out experiments indicate that LIN9 acts as a tumor suppressor by inhibiting DNA synthesis independent of Rb within the G_1_ phase [71]. Tesmin also plays a role in the DNA binding as required by the dREAM complex [72]. 

### Cell Cycle Regulation by MicroRNAs

MicroRNAs are small non-coding ~23nt RNAs that have recently emerged as key post-transcriptional modifiers of gene expression during periods of environmental stress [73-75]. After being transcribed and processed, mature microRNAs are incorporated into the RNA-induced silencing complex (miRISC) to target mRNAs based on sequence complementation in the 3′ untranslated regions (UTRs). These microRNAs are predicted to control the activity of 30-50% of all protein coding genes and have been shown to exert their effects on differentiation, apoptosis, longevity, proliferation and neuroactivity ([74]; Fig. (**6**)). 

MicroRNAs are derived from RNA transcripts that fold into imperfect hairpin structures, classified as primary-microRNAs [76]. Still located within the nucleus, the primary-microRNA transcript is processed by the RNase III type endonuclease, Drosha [77-80]. The Drosha complex processes primary-microRNAs into ~70nt hairpin structures known as pre-microRNA. Drosha processing is necessary to remove introns (mirtrons) that would otherwise interfere with microRNA function [81-83]. Pre-microRNAs are then exported from the nucleus into the cytoplasm, *via *exportin 5, and are then further processed into mature microRNA structures [77-80]. The processing of pre-microRNAs into mature microRNAs is mediated by the endonuclease, Dicer [reviewed in 73,  79, 80, 84].

After processing and formation, mature microRNAs are assembled into microRNA-induced silencing complexes (miRISC). The process of assembly is currently not well understood, but likely involves a dynamic process coupled with the pre-microRNA processing by Dicer [77-80]. Several studies has proposed that the miRISC mediates translational repression *via *binding of the 5’ cap structure of the target mRNA, thereby remodeling and inhibiting translational initiation complexes, while also targeting the mRNA transcript to p-bodies [85]. Similar to the proposed role of microRNAs in the hibernating ground squirrel (*Spermophilus tridecemlineatus*) and frozen wood frog (*Rana sylvatica*), this model provides a process where p-bodies may facilitate long-term mRNA storage; allowing rapid re-initiation of translation of mRNA transcripts immediately after anoxic stress recovery [75, 86]. It is known that the number of p-bodies increase with the onset of environmental stresses, including nutrient deprivation and osmotic stress [87]. This provides an intriguing basis for microRNAs to establish rapid biological controls regulating cell cycle exit during entry into anoxia providing a mechanism by which anoxic turtles can rapidly emerge from a suspended condition and reinstate normal cell cycle activity.

Similarly, results of a study by Dresios suggested that microRNAs (miR-125b) act in response to cold stress [88]. MiR-125b is known to be involved in cell cycle arrest and has been shown to act as a tumor suppressor gene and to regulate cell proliferation in human cancers since one of its targets includes E2F-2 [89]. Although this study does not present the severe temperature extremes or environmental stresses that many other organisms facilitate, it does provide novel and intriguing suggestions that microRNAs may provide an important mechanism in translational repression in response to environmental stresses such as anoxia. 

Fine scale regulation of the cell cycle helps maintain a timely and coordinated progression as well as genetic stability [90]. Specifically, microRNAs targeted by p53 have been shown to increase expression levels during cell cycle arrest. The p53-targeted microRNAs include miR-34a, miR-20a, miR-17-5p, miR-let-7a and miR-615 [91, 92]. Having both experimental and bioinformatic analysis to support their role in the cell cycle, these microRNAs present interesting subjects for further examination (Table **1**). Recent studies show that when cells were transinfected to express high levels of miR-16, an increased number of cells were found to be in quiescence, with corresponding decreases in the numbers of cells in S, G_2_ and M phases [93] Results from these experiments suggest that increased miR-16 expression alone have the ability to arrest cells and promote entry into quiescence.

## 4. OXYGEN DEPRIVATION AND THE MECHANISMS OF CELL CYCLE ARREST

What are the mechanisms for hypoxia induced cell cycle arrest? Researchers have generalized numerous hypoxia sensitive pathways and checkpoints in several organisms, highlighting the multiple interconnected pathways at play and further emphasizing the importance of an appropriate model system. Current studies examining the state of the cell cycle under hypoxic stress have largely been limited to the early developmental stages of brine shrimp (*Artemia franciscana*) embryos, zebra fish (*Danio rerio*) embryos, nematodes (*Caenorhabditis elegans*) and fruit flies (*Drosophila melanogaster*) [3-7]. Research focusing on the developing nematode has found that larval stages can enter a reversible suspended animation in all stages of the cell cycle when presented with an anoxic stress [3, 7]. Similar to research carried out with nematodes, studies utilizing GFP-kinesin in the early embryonic stages of fruit fly development have shown that hypoxia (less than 2% oxygen) induces a prolongation of all cell cycle stages. Furthermore, when exposed to anoxia these embryos enter a reversible arrest at one of two phases: mitosis (aligned, nonsegragated chromatids) and a G_1_-like phase (early embryos do not possess a true G_1_ phase) [7]. Although complete analysis of the cell cycle has not yet been studied, it is known that during the embryonic stages, brine shrimp can enter a quiescent state for several years; a state facilitating the long-term arrest of the cell cycle and allowing for development to resume when environmental conditions become favorable for growth [4, 5, 7]. In the early embryonic stages, zebra fish have been found to enter a state of reversible arrest when presented with an anoxic environment [6]. Flow cytometric studies have indicated that these embryos enter an arrest in the S and G_2_ (4n) phases of the cell cycle. It has been postulated that these phases of the cell cycle may have a higher oxygen demand than other phases [6]. Although there have been several studies characterizing the anoxic response in developing organisms, cell cycle arrest has not yet been characterized for developmentally mature vertebrate organisms tolerant of severe anoxia. 

The study of hypoxia induced cell cycle arrest is complicated by many factors including the requirement of many key players (Cyclins, Cdks and CKIs) and the high degree of redundancy between multiple pathways (such as the Rb and E2F family members). Severe hypoxia is a unique stress as cells undergo a rapid replication arrest without accumulating DNA damage [41, 42]. Hypoxia has also received research attention as it is physiologically relevant, occurring during normal embryogenesis, ischemic injuries and tumor progression [42]. Given the clinical implications pertaining to hypoxia, the proper selection of a model system becomes critical. Relying on model organisms can induce a sense of oversimplification and an overestimate of commonalities directed towards other unexplored species. Ideally, research begins with simple and well-understood organisms and then builds upon this knowledge with wider and more-complex organisms. Problems arise when responses seen in the simple model are taken to represent a complete understanding of a central process, including hypoxic cell cycle arrest. Research carried out with model organisms has proven to be a useful way to analyze cellular processes [94]. However, these organisms have also been subject to several biases. For example, model organisms may be highly available and easy to use; however, there are also restrictions to studying a small number of species and attempting to generalize across a wide range of biological diversity. In order to address these issues in hypoxic cell cycle regulation there needs to be more focus on research carried out in non-model organisms that better facilitate the anoxic stress response pathway.

Anoxia in a vertebrate animal model system, capable of undergoing physiological and molecular reorganization in response to low oxygen, provides an invaluable tool to dissect the anoxic response and proliferation control pathways. One such vertebrate animal capable of surviving extreme reductions in oxygen consumption, and worthy of in-depth exploration at the molecular level, is the red eared slider (*Trachemys scripta elegans*). This turtle has been extensively studied for its ability to suspend breathing and enter an anoxic state, facilitating survival for months at a time. This ability is clearly fascinating and has been the focus of numerous physiological, molecular, and biochemical studies [reviewed in 95,  96].

### The Anoxic Turtle

Turtles comprise a small taxon that has attracted the attention of biologists for centuries. However, despite this attention, a major portion of their life cycle has been left relatively untouched [97]. In their northern ranges, turtles spend upwards of half of their lives in an over-wintering state, the majority of which is located in ice-covered ponds where oxygen levels can drop drastically [97, 98]. Oxygen deprivation is a particularly challenging stress due to its consequences for ATP production, a stress that can rapidly kill intolerant species [99, 100]. The turtle achieves anoxia tolerance primarily through several mechanisms: 1) metabolic rate suppression 2) high capacity for glycolytic energy production including large reserves of substrates (glycogen) and 3) effective methods for dealing with end products (acid buffering, lactate storage in shell). Each of these mechanisms converge on the conservation of ATP stores [95, 101]. 

### Metabolic Rate Depression

Common themes in scientific literature include the associations between metabolic rate, energy status and stress resistance [95, 101]. In many animals, low energy status elicits an altered pattern of gene expression that results in reduced rates of metabolism and is coincident with entry into a quiescent state [95, 102]. Hibernating and/or torpid mammals often suppress their metabolism to less than 10% of that of active animals, whereas anoxic turtles have been known to suppress their metabolic demands to that of 15% of normoxic values [102]. 

Although it is clear that anoxic survival relies on the suppression of ATP consuming processes, the state of the cell cycle in anoxia tolerant organisms remains largely unknown. Proliferation of cells is clearly an energy-expensive biosynthetic process, so it would make sense that these activities are suppressed to a minimum under anoxic conditions. Taking into account that ATP supplies become limited under anoxia stress, in concurrent with reports by Mazia who suggests that withdrawal of energy yielding substances will induce G_1_ arrest if applied before S-phase, a substantial G_1_/G_0 _arrest during anoxia seems highly probable [103]. The demanding energetics of mitosis provides an intriguing suggestion that the cell cycle may arrest in proliferating tissues in order to facilitate metabolic rate depression and play an important role in cellular responses to environmental stresses. 

Indeed, studies with other systems of facultative metabolic arrest, such as mammalian hibernation, do induce cell cycle arrest in hypometabolic states. For example, studies with hibernating ground squirrels (*Crypturellus undulatus*) indicate that highly regenerative tissues such as intestinal epithelial are suspended in the 2N DNA range (G_1_/G_0_) (90%) during hibernation as compared to active states (79%); whereas, both S and G_2_/M phases are decreased during hibernation [104, 105]. These results suggest that DNA synthesis is markedly reduced during the hibernation period. As previously mentioned, in other species with varying degrees of stress tolerance, sites of cycle arrest appear to differ. However, a number of DNA replication checkpoint proteins have been shown to be involved in general hypoxic cycle arrest, and some have been suggested to transduce the stress signal [7]. These include Cdk 1, Cdk 2, Cdk 4, Cdk 6, Cyclins A, D, and E, Rb pocket proteins and specific CKIs (specifically p21, p27, and p16) [12, 19, 21, 29, 36, 41, 58].

Current research in our lab is focused towards the biochemical adaptations that support survival of anoxia by the turtle, *T.s.elegans*. In summary, the mechanisms of cell cycle arrest could contribute substantially to an overall reduction in energy consumption in the anoxic state. Arrest of cellular proliferation should be crucial for ATP homeostasis in the anoxic state, since aberrant continuation of the cell cycle would lead to rapid depletion of energy stores. 

Activation of an Rb:E2F mediated quiescent phase during periods of severe stress is a common response in hypoxia tolerant organisms, such as *C.elegans* and *D.melanogaster* [7, 14]. Therefore, it is important to learn how developmentally mature vertebrate systems tolerant avoid energy depletion and whether this is accomplished through the evolutionarily conserved mechanism of Rb:E2F mediated G_1_ cell cycle arrest. The effect of anoxia on Rb phosphorylation and the hypophosphorylated state found in many hypoxia tolerant organisms may perhaps be a mechanism by which cells exit the cell cycle in G_1_ or G_0_ quiescence arrest, mediated in part by many of the typical stress response pathways discussed in this review. The effect of anoxia on other important proteins involved in G_1_/S transition, which include E2F and Cyclins D and E are not yet defined. Furthermore, kinases such as ATM, and its downstream effector Chk2, may be crucial for the sensing and implementation of anoxia-responsive transition into cell cycle arrest. 

Furthermore, long term oxygen deprivation may facilitate a new and novel mechanism of cell cycle arrest, distinct from nematodes, brine shrimp, fruit flies and zebra fish. Examination of the key mechanisms discussed in the review will provide a critical overview and elucidation of the main mechanisms facilitating cell cycle arrest and direct future studies in ischemic injury and tumor growth. 

Clearly, *T.s.elegans* provides an excellent model system for studying the process of anoxic cell cycle arrest, and the current research provides many new and exciting questions regarding both generalized processes and mechanisms of arrest. Many studies focused on cell cycle arrest examine the mechanisms of arrest in invertebrate or vertebrate embryo model organisms. These studies provide a solid foundation for further studies, but do not highlight the complexities of developed vertebrate life as would studies on the anoxic turtle. Research on the anoxic turtle will most certainly demonstrate the cellular and molecular mechanisms underlying the response to anoxia and the control of cellular proliferation. These studies will also provide a wide comparative data from biologically diverse organisms and highlight the essential processes eliciting cell cycle control in an anoxic environment. Discovering the commonalities of response pathways in organisms as diverse as nematodes, fruit flies, and turtles will undoubtedly lead to refined treatment of ischemic injuries and to hypoxic tumor cores which are often resistant to radiation and chemotherapy [106].

## Figures and Tables

**Fig. (1):**
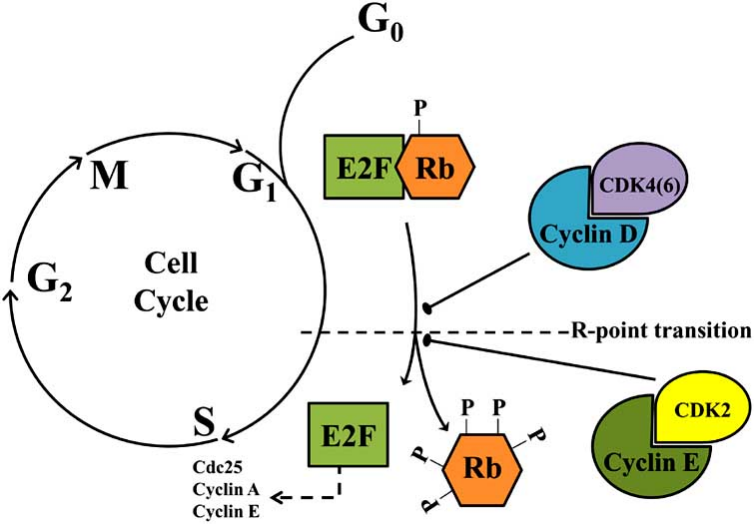
The Rb:E2F pathway. Sequential phosphorylation by kinase complexes Cyclin D:Cdk 4/6 and Cyclin E:Cdk 2, respectively, causes conformational changes to the Rb structure and release of E2F. The release of E2F is necessary for the expression of S-phases genes.

**Fig. (2):**
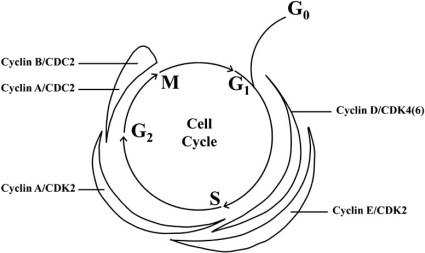
Expression profiles of Cyclin:Cdk complexes throughout the cell cycle. Cyclic expression of these complexes allow for the completion of one phase before the initiation of the subsequent phase [107].

**Fig. (3):**
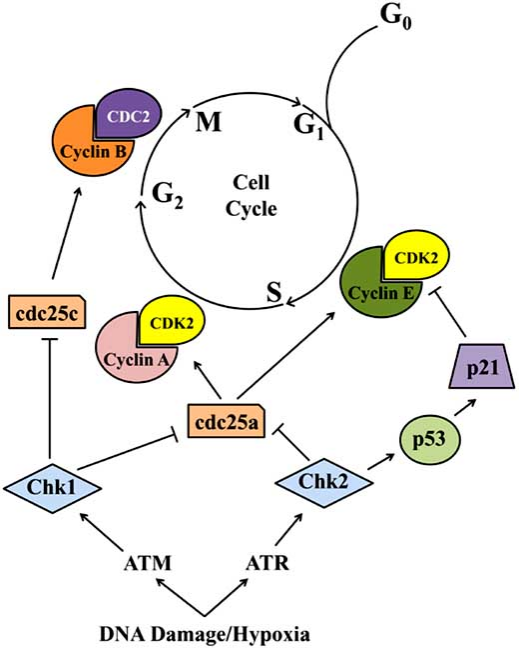
The ATM/ATR DNA damage response pathway and its downstream effectors leading to either G_1_/S or G_2_/M phase arrest.

**Fig. (4):**
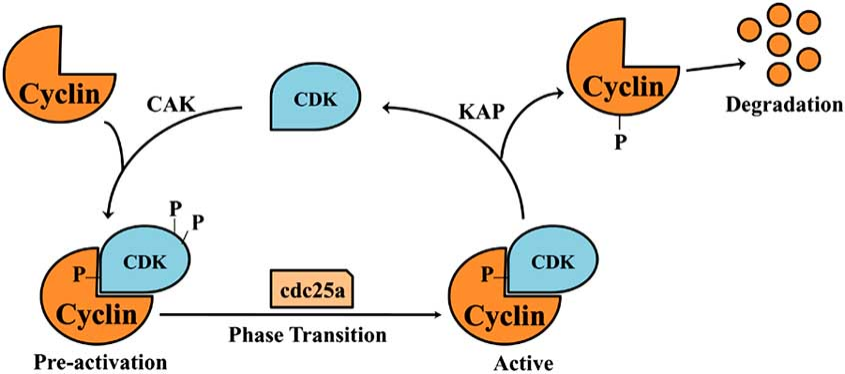
Mechanism of Cdk activation involving regulatory phosphorylation and Cyclin binding.

**Fig. (5):**
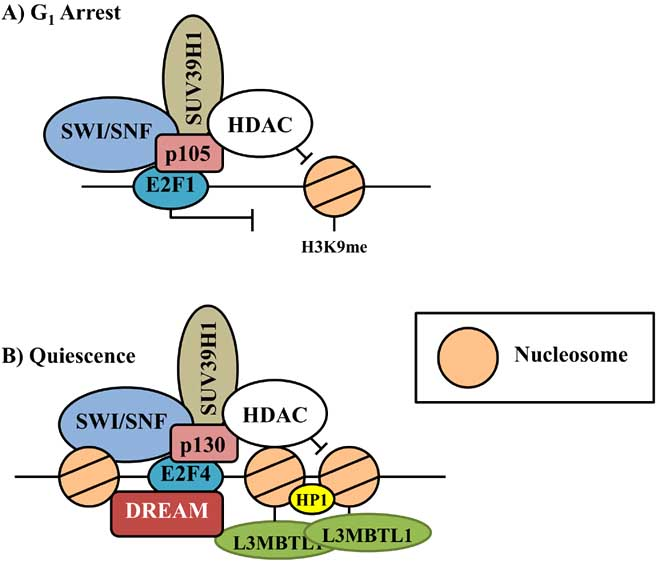
Chromatin remodeling complexes associated with the Rb family. Complex composition changes with the duration and type of cell cycle arrest. Pictured are the complex members in **A**) cells undergoing general G_1_ arrest and **B**) cells undergoing a reversible exit from the cell cycle (quiescence) [108].

**Fig. (6):**
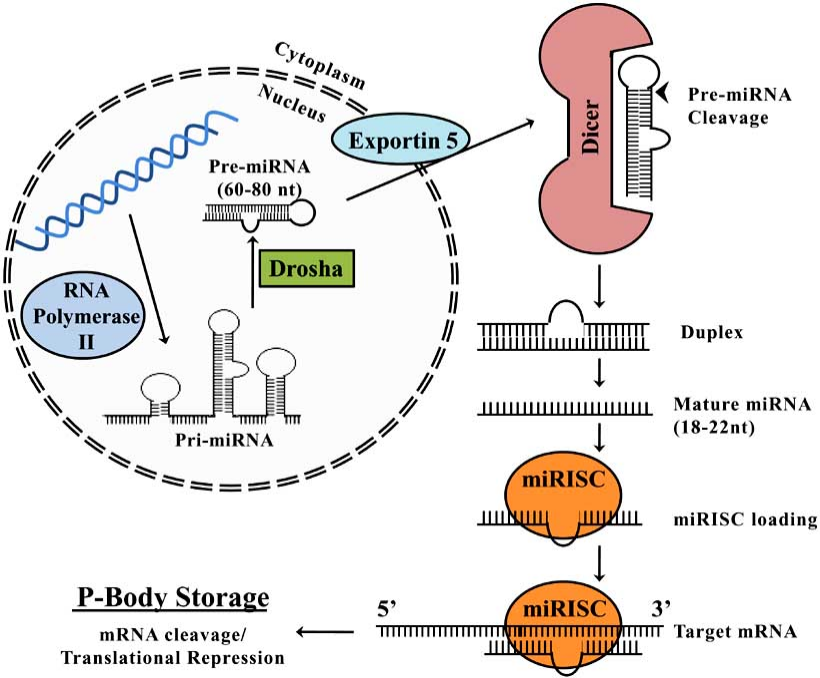
MicroRNA biogenesis. Primary transcripts are transcribed by RNA polymerase II and excised by a series of riboendonucleases into single-stranded mature microRNA stuctures. Mature microRNA structures are then loaded into the microRNA induced silencing complex (miRISC) and represses translation of targeted mRNA through 3- UTR binding.

**Table 1:** Key Regulatory Cell Cycle Components Experimentally Determined to be Targeted by microRNAs

MicroRNA	mRNA Target	References
miR-34a	E2F-3, Cyclin D and Cdk 6	[109, 112]
miR-16/miR-15	Cdk 4, Cyclin D & E, cdc25a	[93, 112]
miR-107	Cdk 6, cdc25a, Cyclin E, E2F-1	[110]
miR-20a	Cdk 2, E2F-1 and Cyclin A	[111, 112]
miR-17-5p	E2F-1 and Cyclin A	[111]
miR-let-7a	cdc25a and Cdk 6	[112]
miR-148a	E2F-3	[113]
miR-103	Cyclin E, cdc25a, Cdk 6, E2F-1	[114]
